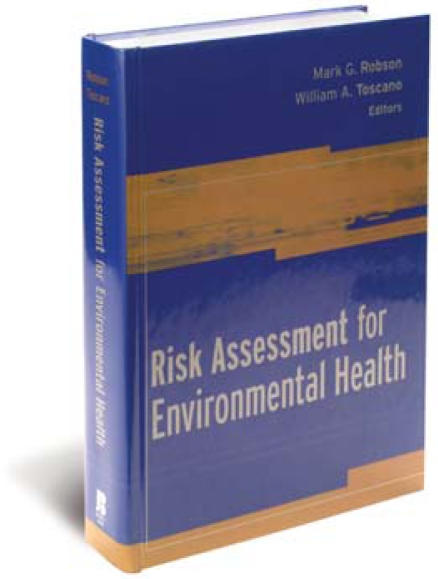# Risk Assessment for Environmental Health

**Published:** 2008-12

**Authors:** Reuben Thomas

**Affiliations:** Reuben Thomas is a Visiting Fellow working under the supervision of Christopher Portier in the Environmental Systems Biology group at the National Institute of Environmental Health Sciences. He obtained his PhD. in computational biology from Northwestern University in 2007. His research deals with use of mathematical and statistical techniques in biological networks

Edited by Mark G. Robson and William A. Toscano.

San Francisco:Jossey-Bass, 2007. 628 pp. ISBN: 978-0-7879-8319-2, $85.

This comprehensive and easy-to-read treatment of the various facets, history, and criticisms involved in risk assessment for environmental health focuses on tools and approaches used for humans in an environment involving potential chemical hazards.

The first part introduces the underlying principles and techniques of the field, and the second examines case studies in terms of different risk assessment scenarios. I would have preferred a finer division of the book (with chapters in a different sequence).

The first three chapters introduce risk assessment and place it in the broader context of how risk is managed and how risk characterizations must be communicated effectively to different stakeholders. Chapter 14 describes the history of managing risk by acts passed by the federal and state governments, as well as the U.S. government system that allows for these enforcements. Chapter 16 presents the various steps and issues involved in risk communication. The notion of involving the broader community in the process of prioritizing risks from different sources in the environment is explained in the chapter on comparative risk assessment (Chapter 8) and illustrated with the help of the Ohio comparative risk project case study (Chapter 26). The authors of Chapter 15 argue that the current paradigm of risk assessment/management must be revised to minimize harm as quickly as possible. Using the precautionary principle a completely proven causal link to risk would be replaced by a search for alternatives when there is a somewhat dependable hint of substantial risk. Chapter 4 describes the principles of toxicokinetics and toxicodynamics and justifies the steps defined for risk assessment from a toxicologic viewpoint. In light of the bias of chemical testing for carcinogenicity potential, a section in this chapter is devoted to the current theories of carcinogenesis.

Various computational and experimental tools are described in Chapters 5, 6, 7, and 13, and their application is demonstrated in the case studies described in Chapters 17, 20, and 33. Physiologically based pharmacokinetic (PBPK) models used to model the absorption, distribution, metabolism, and excretion of the chemical of interest are described in Chapter 5, including the models’ uncertainty and variability. Basic mathematical equations that capture risk assessment notions are introduced in Chapter 6, along with ways of considering multiple exposures and different routes for the same exposure and Monte Carlo methods to quantify uncertainties in the model predicted responses. Modern molecular measurements involving genes, proteins, and metabolites are described in Chapter 7. The variability between individuals is illustrated by the presence of single nucleotide polymorphisms for genes governing the metabolism of xenobiotic chemicals. Chapter 13 describes the various issues and techniques involved in collecting biological samples for monitoring the presence of chemicals.

Other chapters describe risk assessments that differ from the standard approach: assessment involving microbial or radiological sources, susceptible populations such as children, and chemicals that have an optimum range of no effect. The history and the current state of regulating the working conditions of workers are also presented, including disparities in standards between the workforce and the general community.

The book emphasizes the need for risk assessment, the inherent uncertainties in arriving at a quantitative estimate of risk, and the value judgments, technology, commerce, and politics that play important roles in the final enforced risk standard. Also emphasized are multiple routes of exposure and the need for special attention to risk assessments for susceptible populations such as children.

Overall, the book reads less like a standard textbook and more like a reference source. Material discussed in one part of the book is again discussed in another part without the addition of anything new. For example, the PBPK model of methylene chloride is discussed in at least three parts of the book in different contexts but the descriptions do not reference the others. Each chapter, helpfully, begins with a list of summary points and ends with questions that require the student to think beyond and to access resources outside the book. The figure captions could have been more descriptive, and color would have made some of the figures easier to decipher. Finally, for the curious student, some portions of the book are not self-contained and would require reading from additional references.

## Figures and Tables

**Figure:**